# Parkinsonism caused by adverse drug reactions: a case series

**DOI:** 10.1186/1752-1947-5-105

**Published:** 2011-03-16

**Authors:** Solomon O Ugoya, Emmanuel I Agaba, Comfort A Daniyam

**Affiliations:** 1 Department of Medicine, Jos University Teaching Hospital, PMB 2076, Jos, Nigeria

## Abstract

**Introduction:**

Parkinsonism puts a high direct cost burden on both patient and caregiver. Several reports of drug-induced parkinsonism have been published, but to the best of our knowledge, there has not been any report of quinine or halothane inducing parkinsonism.

**Case presentation:**

We describe two cases of parkinsonism possibly caused by adverse drug reaction to quinine in a 29-year-old black Nigerian woman and to halothane in a 36-year-old black Hausa (Nigerian) man who received it as general anaesthesia for appendicectomy in our teaching hospital.

**Conclusion:**

These are two unusual cases of parkinsonism caused by adverse drug reactions to high-dose quinine and to halothane as general anaesthesia. We consider that these two cases are important in bringing this potential side-effect to the attention of both pharmacologists and primary care physicians as these are two of the most commonly used medications in our clinics. We conclude that parkinsonism should be included among the adverse drug reactions to high-dose quinine and halothane general anaesthetic.

## Introduction

The most common cause of parkinsonism is Parkinson's Disease (PD), accounting for approximately 77.7% of cases, followed by parkinsonism-plus syndrome (12.2%). Secondary causes such as drugs, trauma, vascular conditions, acquired immunodeficiency disease and toxins make up around 8.2%, and the remaining 0.6% of cases are classified as Heredodegenerative parkinsonism [1].

Quinine was the first antimalarial drug available, which has been in use since the 17th century [2]. Quinine is a natural white crystalline alkaloid with antipyretic effects. It is a stereoisomer of quinidine, which has an antiarrhythmic effect. Side effects of quinine include cinchonism, pulmonary oedema, abnormal heart rhythms and hearing impairment [3]. Contrary to popular belief, quinine is an ineffective abortifacient, and is recommended by World Health Organization (WHO) for use in pregnancy [4]. The mechanism of action of quinine is still elusive but the most popular hypothesis is inhibition of hemozoin biocrystallization leading to formation of cytotoxic heme, which accumulates within the malaria parasites and causes their death [5].

Halothane is an inhalational anaesthetic agent, chemically designated 2-bromo-2-chloro-1,1,1-trifluoroethane. Halothane anaesthesia augments the action of nondepolarizing skeletal muscle relaxants and ganglionic blocking agents, and is also a potent uterine relaxant [6]. The side effects include hepatic necrosis, cardiac and respiratory arrest, hypotension, cardiac arrhythmias, hyperpyrexia, shivering, nausea and emesis [6].

Several drugs and toxins have been reported to cause parkinsonism, but to the best of our knowledge, there has been no report of parkinsonism induced by quinine or halothane. The aetiology of PD is still elusive, although a combination of environmental and genetic factors is implicated [7]. The anatomy of the basal ganglia consists of group of nuclei situated in the deep part of the cerebrum and upper part of the brain stem. The neurophysiological changes in the basal ganglia responsible for parkinsonism are mainly neuronal loss in the substantia nigra with consequent dopamine depletion in the striatum [8]. Evidence is accumulating about mechanisms by which drugs and toxins cause parkinsonism. They act on particular steps in the formation, storage in presynaptic vesicles, release, uptake, catabolism and re-synthesis of neurotransmitters such as dopamine, serotonin, norepinephrine, acetylcholine and other catecholamines [9].

## Case presentations

### Patient 1

A 29-year-old Nigerian Edo woman, presented with a five-day history of tremor of her fingers and head at rest, with associated drooling of saliva from her mouth. There was no history of fever, vomiting, headache, dyspeptic symptoms or any other neurological symptoms, although she reported dizziness. She had not used any drugs in the preceding six weeks except for quinine tablets. She reported having had amenorrhea for eight weeks, and believing that she was pregnant, she had taken some tablets of quinine to terminate the pregnancy. She reported having vaginal bleeding five days after ingestion of six tablets of quinine. She had no family history of PD.

On physical examination, our patient was found to have mask-like facies, and her speech was monotonous and slightly slurred. She was extremely slow in carrying out all motor activities. Results of neurological examinations were essentially normal except for the presence of cogwheel and global muscular rigidity. Results of blood chemistry and haematological studies were normal, and serum syphilis and human immunodeficiency virus (HIV) testing were negative. Chest radiography and brain magnetic resonance imaging were essentially normal. A pregnancy test was positive and a pelvic ultrasound scan showed a bulky uterus with product of conception.

Based on the clinical signs and symptoms, a diagnosis of drug-induced Parkinsonian syndrome was made. We started our patient on low-dose levodopa/carbidopa 100/25 mg tablets twice daily. After five days of treatment, all the symptoms had disappeared. The patient recovered completely and had no recurrence of symptoms during follow-up of 30 weeks after discontinuing treatment.

### Patient 2

A 36-year-old Nigerian Hausa man who had undergone an appendicectomy was referred to our neurology unit with tremor at rest and bradykinesia. The surgery had been uneventful, and our patient had not taken any medication likely to affect the central nervous system for six weeks before his surgery. His only medication had been the halothane that was used as general anaesthetic for his appendicectomy, along with preoperative medications consisting of 5 mg diazepam injections, 1 mg starting dose of atropine, and 50 mg of pethidine. Postoperatively, he received intravenous ampicillin and cloxacillin at a dose of 1 g every six hours for one week, and 50 mg of tramadol every 12 hours for five days. He has no family history of PD.

A careful neurological evaluation revealed asymmetrical rigidity, affecting the right side more than the left, with hypokinesia and tremor. Laboratory investigations, including full blood count, electrolytes and urea, and testing for malaria parasite, HIV and syphilis were essentially normal.

Our patient was diagnosed with drug-induced parkinsonism (DIP) and started on levodopa/carbidopa (250 mg) daily. After three days of treatment, the symptoms resolved completely and six weeks after he remained healthy.

## Discussion

Drugs are the most common causes of parkinsonism in the general population, and are generally drugs that block postsynaptic dopamine receptors or deplete presynaptic dopamine. Several drugs have been implicated as a cause of parkinsonism, including dopamine antagonists such as prochlorperazine for giddiness, metoclopramide for dyspepsia and chlorpromazine for bipolar depression; calcium-channel blockers such as flunarizine and Cinnarizine; and sodium valproate for epilepsy and migraine headache. Some of these medications may also be adulterated because of increased access via the internet [10].

Chabolla *et al*. reported that DIP is indistinguishable from PD [11]; however, Susatia and Fernandez contradicted this in their recent review, suggesting that DIP can be distinguished from PD because tremor and gait instability are less prominent in DIP [12]. Treatment of DIP involves discontinuation of the offending drugs, which usually promotes remission of the Parkinsonian syndrome within a short time, although parkinsonism may sometimes persist. Such patients may have subclinical parkinsonism and require dopaminergic treatment as with our two patients. PD is usually irreversible, but DIP is reversible [13]. MRI scans usually give normal results in DIP, as in our first patient; our second patient could not afford to undergo neuroimaging studies.

The clinical findings in our two patients are unusual adverse reactions of quinine or halothane administration. The response to levodopa was an indication of loss of dopamine in the dopaminergic systems, which may explain the remarkable effects of levodopa in these two cases.

The effects of general anaesthesia were for many years attributed to changes in the physical chemistry of neurons. More recently, it has been linked to ligand-gated ion channels and to alterations in neurotransmitter function. Inhalational anaesthesia has been known to inhibit brainstem activity to the extent that pupillary reflexes and corneal reflex is eliminated with complete return to normal by the time the patient has recovered sufficiently to talk [14]. Nevertheless, Adachi *et al*. were able to show that halothane causes decrease in dopaminergic activity in a rat brain model [15].

The mechanism by which inhalational general anesthesia acts on the brain is still unknown. It has been assumed that the lipid solubility of these compounds alters the physicochemical properties of the cell membrane and thus impairs the activities of the membrane receptor systems that regulate ion channels, particularly the chloride and calcium channels [16]. However, Freedman *et al*. have shown that quinine blocks the K+ channels in rat corpus striatum and that this blockade may be attained in human brain at an antimalarial dosage of quinine [17].

## Conclusion

In conclusion, the side effects of quinine and of inhalational anaesthetics such as halothane can now be broadened to rarely include parkinsonism, and should be considered in patients presenting with similar symptoms. As with other symptoms more commonly associated with this condition, these may resolve after discontinuation of the offending drugs or administration of levodopa for persistent symptoms. Further studies are warranted to elucidate the relationship and the mechanism by which halothane and quinine can cause parkinsonism.

## Competing interests

The authors declare that they have no competing interests.

## Authors' contributions

US was responsible for the study concept and design and the acquisition of data, and supervised the study. US and AI drafted the manuscript, and US, AI and DC were responsible for critical revision of the manuscript. US, AI and DC provided administrative, technical and material support. All authors read and approved the final manuscript.

## Consent

Written informed consent was obtained from the patients for publication of this case series and any accompanying images. Copies of the written consent are available for review by the Editor-in-Chief of this journal.
